# Low oral dose of 4-methylumbelliferone reduces glial scar but is insufficient to induce functional recovery after spinal cord injury

**DOI:** 10.1038/s41598-023-46539-5

**Published:** 2023-11-06

**Authors:** Kateřina Štepánková, Milada Chudíčková, Zuzana Šimková, Noelia Martinez-Varea, Šárka Kubinová, Lucia Machová Urdzíková, Pavla Jendelová, Jessica C. F. Kwok

**Affiliations:** 1 Institute of Experimental Medicine, Czech Academy of Sciences, Vídeňská, 1083 Prague, Czech Republic; 2 Department of Neuroscience, Charles University, Second Faculty of Medicine, 15006 Prague, Czech Republic; 3 Institute of Physics, Czech Academy of Sciences, 182 21 Prague, Czech Republic; 4 Faculty of Biological Sciences, University of Leeds, Leeds, LS2 9JT UK

**Keywords:** Molecular neuroscience, Regeneration and repair in the nervous system, Synaptic plasticity

## Abstract

Spinal cord injury (SCI) induces the upregulation of chondroitin sulfate proteoglycans (CSPGs) at the glial scar and inhibits neuroregeneration. Under normal physiological condition, CSPGs interact with hyaluronan (HA) and other extracellular matrix on the neuronal surface forming a macromolecular structure called perineuronal nets (PNNs) which regulate neuroplasticity. 4-methylumbelliferone (4-MU) is a known inhibitor for HA synthesis but has not been tested in SCI. We first tested the effect of 4-MU in HA reduction in uninjured rats. After 8 weeks of 4-MU administration at a dose of 1.2 g/kg/day, we have not only observed a reduction of HA in the uninjured spinal cords but also a down-regulation of CS glycosaminoglycans (CS-GAGs). In order to assess the effect of 4-MU in chronic SCI, six weeks after Th8 spinal contusion injury, rats were fed with 4-MU or placebo for 8 weeks in combination with daily treadmill rehabilitation for 16 weeks to promote neuroplasticity. 4-MU treatment reduced the HA synthesis by astrocytes around the lesion site and increased sprouting of 5-hydroxytryptamine fibres into ventral horns. However, the current dose was not sufficient to suppress CS-GAG up-regulation induced by SCI. Further adjustment on the dosage will be required to benefit functional recovery after SCI.

## Introduction

Spinal cord injury (SCI) is a damage to the spinal cord that causes partial or complete loss of control of locomotor and sensory functions^1^. SCI itself is a dynamic process starting immediately after the injury when tissue damage is continued with haemorrhage, inflammation, as well as oedema. The acute phase includes initial trauma followed by spinal cord ischemia, cell excitotoxicity, ion dysregulation, and free radical-mediated peroxidation^2^. These processes initiate a complex secondary injury cascade leading to changes in structural architecture of the spinal cord characterised by the chronic phase. Chronic phase begins 6 months after SCI in human and continues throughout the lifetime of the patient. It is characterized by the stabilization of the lesion including scar formation accompanied by alterations in neural circuitries^3^. As there is no successful regenerative treatment available, most patients remain in the chronic state for the rest of their life.

Adult central nervous system (CNS) has relatively poor regeneration capacity caused by both the extrinsic inhibitory environment after injury (such as glial scar and myelin-associated inhibitors) and the intrinsic poor regeneration ability of the neurons themselves^4,5^. While these extrinsic and intrinsic factors co-ordinate to maintain the stability of the neural networks in a healthy state, they rapidly become the major obstruction to regeneration post-injury^6^. Perineuronal nets (PNNs) are extracellular matrix (ECM) structures enwrapping a sub-population of neurons in the CNS and play a crucial role in plasticity regulation during postnatal development and post traumatic regeneration^7,8^. PNNs form at the end of the critical period in the CNS and terminate developmental neuroplasticity. PNNs mature by early adulthood^9^ and stabilize synaptic contacts and are dynamically maintained during the lifespan^9–11^. In spinal cord, PNNs mainly surround motoneurons in the ventral horns^12–14^ that are directly responsible for the motor activity^15^. PNNs are composed of a multitude of neural ECM components where chondroitin sulfate proteoglycans (CSPGs) and hyaluronan (HA) are the major constituents^8,16,17^.

Degradation of PNNs reactivates juvenile-like state of plasticity which enables axon sprouting and regeneration of function by synaptogenesis and experience-dependent synaptic plasticity after SCI^18^. Enzymatic removal of PNNs through chondroitinase ABC (ChABC), that digests chondroitin sulfate glycosaminoglycan (CS-GAG) chains on CSPGs, has shown to reopen a critical window for plasticity enhancement and promote functional recovery in multiple SCI models^19,20^. Moreover, when ChABC is combined with rehabilitation, the effect of ChABC treatment is enhanced as compared to non-rehabilitating animals^21,22^. CSPG synthesis inhibitors, such as fluorosamine and its analogues, have also been shown to reduce CS-GAG level and accelerate remyelination following focal demyelination in mice^1,23^. These results suggest that CSPG synthesis inhibitors are possible approaches to enable axonal regeneration after CNS injury.

We have recently reported the use of a small molecule 4-methylumbelliferone (4-MU) to down-regulate PNNs and re-activate neuroplasticity for memory enhancement^24^. 4-MU is a known inhibitor to HA synthesis^25,26^. Previous work has shown that 4-MU inhibits the production of UDP-glucuronic acid (UDP-GlcA)^27^, a key substrate for HA production, and the expression of hyaluronan synthases (HASs), UDP-glucose-pyrophosphorylase (an enzyme for the biosynthesis of UDP-glucose) and UDP-glucose-dehydrogenase (an enzyme that converts UDP-glucose into UDP-glucuronic acid)^28,29^. Interestingly, UDP-GlcA is also a substrate for the synthesis of CS, as well as some other GAGs including dermatan and heparan sulfates. The effect of 4-MU in general GAG synthesis has yet to be clarified in vivo.

Our previous observation of PNN down-regulation by 4-MU prompts us to ask the question if 4-MU would be effective in down-regulating the inhibitory ECM (i.e. ECM molecules, such as proteoglycans, which are up-regulated after injury and restricting axonal growth) in the glial scar after SCI. Here, we aimed to evaluate the biochemical and histological changes in the injured spinal cord after long-term 4-MU treatment in rats and to evaluate the effect of this molecule on anatomical changes in the spinal cord after chronic SCI. Histological staining of PNNs using *Wisteria floribunda agglutinin* (WFA) and anti-aggrecan antibody (ACAN, a CSPG) shows PNN down-regulation in 4-MU-treated group, and that the staining recovers in wash-out group. We also observed that the long-term 4-MU treatment leads to a significant reduction in the glial scar and promotes the sprouting of serotonergic fibres above and below the lesion.

## Results

### 4-MU decreases GAG synthesis and PNN in uninjured spinal cord

**Figure 1:**
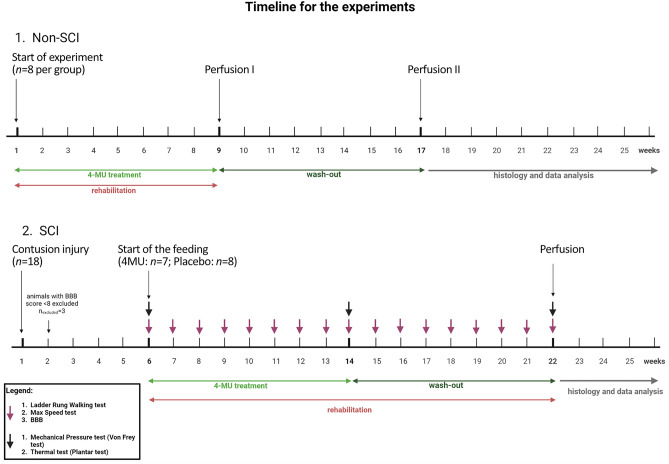
Schematic illustration of the experimental timeline. Created with BioRender.com.

We first investigated the effect of 4-MU in the down-regulation of GAGs in the CNS and its effect on PNNs, with and without treadmill training which is a common rehabilitation. We tested the efficacy of a lower dose at 1.2 g/kg/day weight aiming to minimize the 4-MU consumption for potential adverse effects of long-term treatment for SCI^30^. A timeline for the feeding regime can be found in Fig. 1 (Non-SCI).

**Figure 2:**
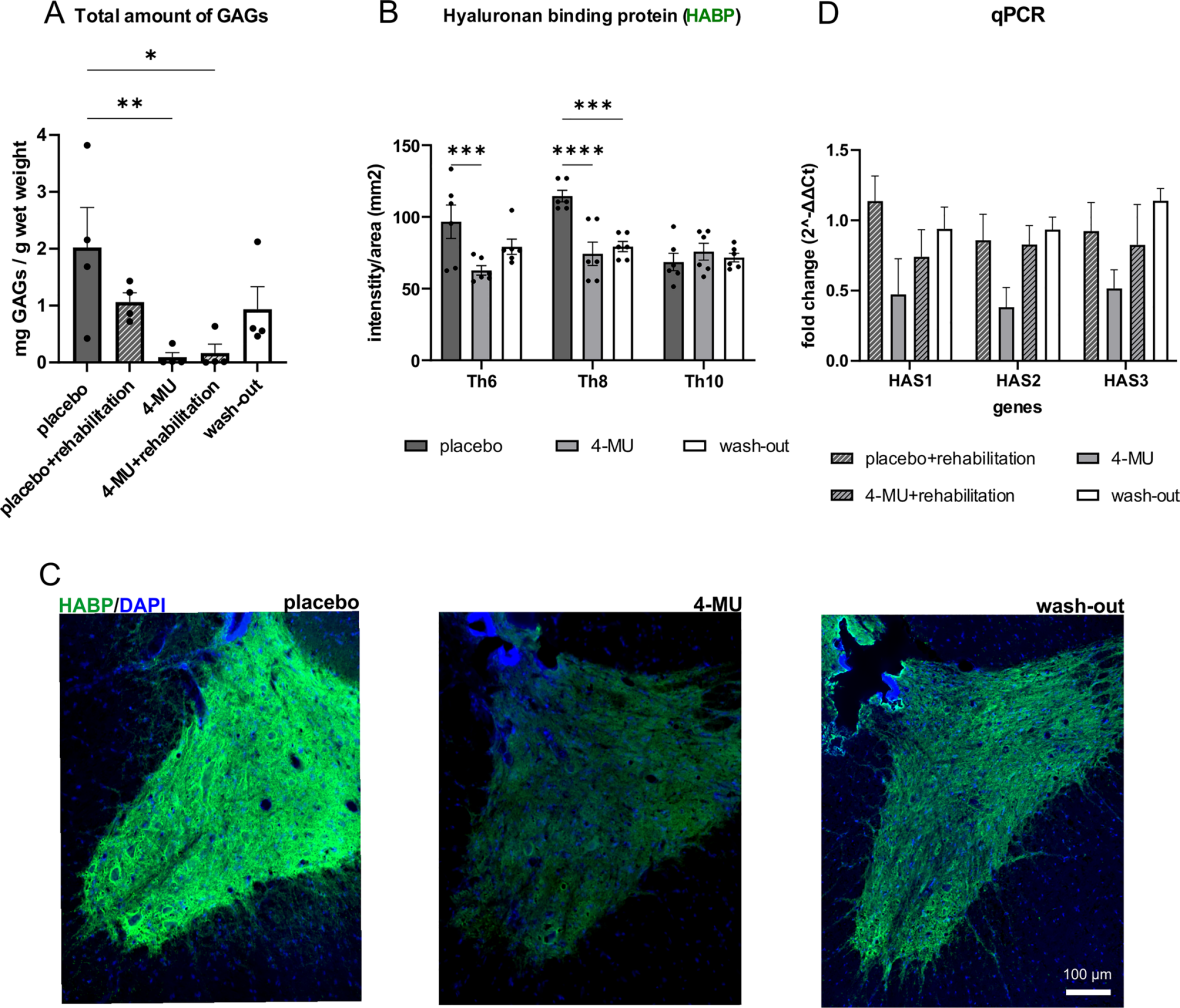
4-MU decreases hyaluronan (HA) and chondroitin sulfate proteoglycans (CSPGs) synthesis in non-SCI animals. (**A**) Bar graph showing the total amount of glycosaminoglycans (GAGs) extracted from frozen spinal cords after 4-MU or placebo feeding, and wash-out period. Values are plotted as mean ± SEM; * *p* < 0.05, by one-way ANOVA, Dunnett's *post-hoc* test. (*n* = 4 animals per group). (**B**) Quantification of (C). Bar graph shows the mean intensity per area of grey matter together with the individual data points. Intensity was calculated using the HistoQuest software from TissueGnostics. Values are plotted as mean ± SEM; *p < 0.05, **p < 0.01 by two-way ANOVA, Dunnett's *post-hoc* test. (*n* = 3 animals per group, 2 images per animal). (**C**) Representative fluorescent images showing different hyaluronan binding protein positive (HABP +) signal intensity in gray matter (Th8) in placebo, 4-MU treated and wash-out group after 8 weeks of feeding and after 2 months of wash-out period. Scale bar 100 µm. (**D**) Bar graph shows fold changes of the expression of *hyaluronan synthase* (*HAS*) *1, 2, 3* genes (as 2^-ΔΔCt), normalised to the placebo treated animals without rehabilitation. 2^-ΔΔCt values were determined by qRT-PCR. Values are plotted as mean ± SEM; two-way ANOVA, Tukey *post-hoc* test in all 4 groups. (*n* = 4 animals per group).

Adult rats were fed with 4-MU at a dose of 1.2 g/kg/day daily for 8-weeks, and some of them were then subjected to 2 months of wash-out. We extracted the GAGs from the dissected spinal cords and quantified the total amount for HA and GAGs using turbidity assay^31^ (Fig. 2A). The results showed that 4-MU treatment alone (0.09 ± 0.81 mg/g; *n* = 4; *p* = 0.0086) and 4-MU plus daily treadmill training (0.17 ± 0.16 mg/g; *n* = 4; *p* = 0.0113) have significantly reduced the level of GAGs compared to placebo group (2.02 ± 0.70 mg/g; *n* = 4). Rehabilitation did not affect the effectiveness of 4-MU in down-regulating GAG synthesis in treated animals. Interestingly, daily treadmill training alone also showed a modest, but non-significant reduction in GAGs level when combined with placebo (1.06 ± 0.16 mg/g; n = 4; *p* = 0.2555) suggesting that rehabilitation (or training) can independently reduce the level of GAGs. In wash-out group, the total amount of GAGs (0.93 ± 0.40 mg/g; *n* = 4; *p* = 0.1734) recovered to a level similar to the rehabilitating group, suggesting a partial return of GAGs (Fig. 2A). 4-MU and/or rehabilitative effect on GAGs level was tested against placebo group.

We also quantified the level of HA down-regulation using HABP staining (Fig. 2B,C). Histochemical staining was performed on sections at Th6 and Th10 and around the Th8 level which is the focus of subsequent experiments. In 4-MU treated group, the intensity of HABP was significantly decreased at Th8 (74.32 ± 12.81; *n* = 3; *p* < 0.0001) and Th6 sections (68.68 ± 4.20; *n* = 3; *p* = 0.0007) when compared to placebo group (at Th8, 114.53 ± 6.36, *n* = 3 and at Th6, 114.57 ± 10.97, *n* = 3). There was no significant difference between 4-MU-treated and placebo groups at Th10 level. After 2 months of wash-out, HA remained at low levels at Th8 (79.37 ± 5.57; *n* = 3; *p* = 0.0005) and Th6 (83.24 ± 11.00; *n* = 3; *p* = 0.0962) compared to the placebo group, with some trend of return of HA production but not reaching the level of the placebo group (Fig. 2B,C).

Quantification of the mRNA expression of HASs in spinal cord samples using qPCR (Fig. 2D) revealed no significant down-regulation of *HAS genes* expression in the 4-MU group. However, clear trends of down-regulation were observed for *HAS genes* expression in 4-MU group when compared to all other groups. In the wash-out group, the *HAS genes* expression reached placebo levels combined with rehabilitation group, suggesting a recovery of normal GAGs expression after the wash-out period.

**Figure 3:**
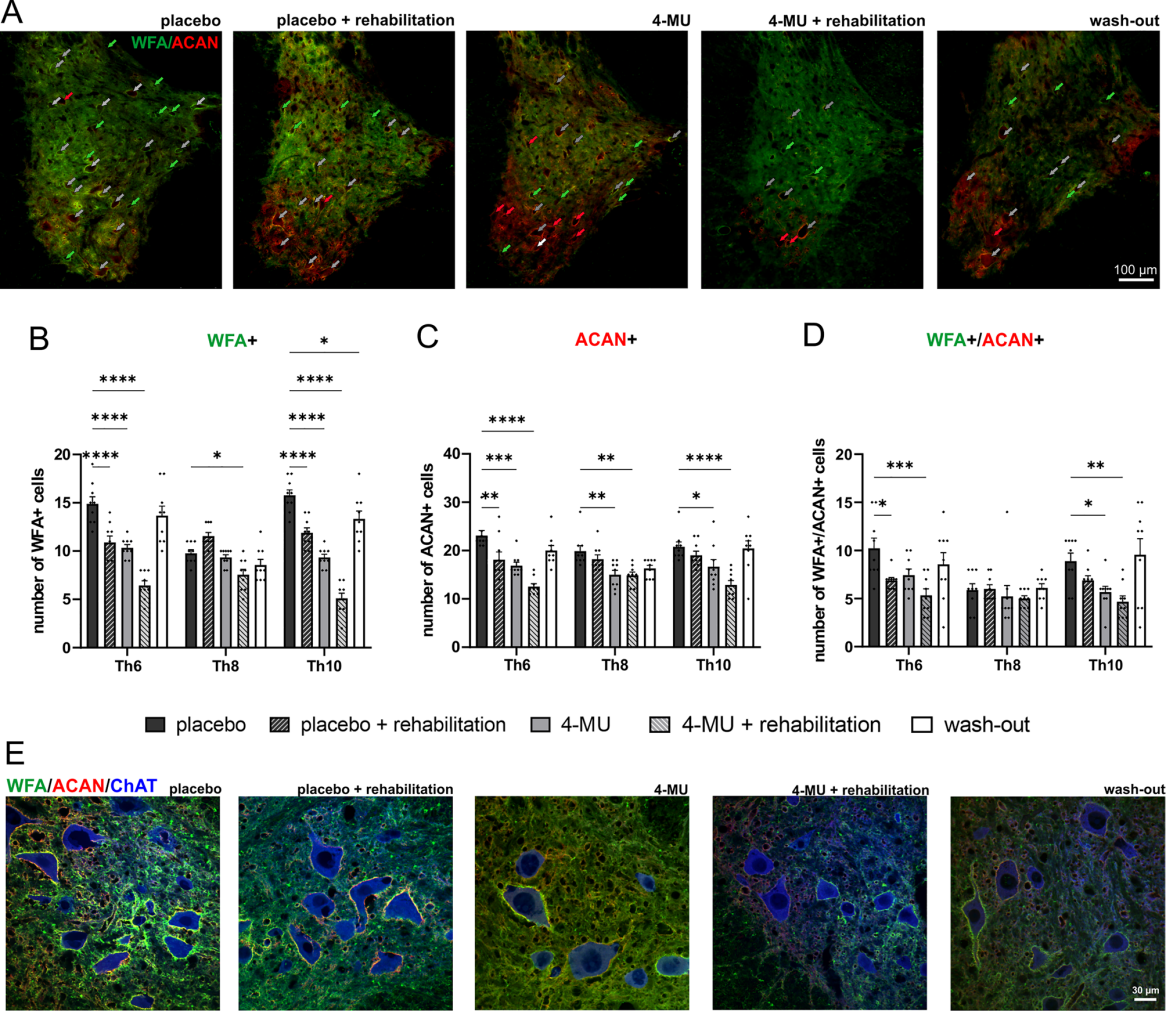
Down-regulation of perineuronal nets (PNNs) after 8 weeks of 4-methylumbelliferone (4-MU) feeding in uninjured animals, with or without daily treadmill training, and the re-appearance of perineuronal nets (PNNs) after 2 months of wash-out period. (**A**) Representative fluorescent images showing *Wisteria floribunda* agglutinin (WFA) and aggrecan (ACAN) positive PNNs around cells in the ventral horns and their colocalization (WFA + /ACAN +) in thoracic spinal cord (Th8) in uninjured animals in all 5 groups after 8 weeks of feeding and after 2 months of wash-out period. Green arrows indicated the WFA positive PNNs enwrapped cells; red arrows indicated ACAN positive PNNs enwrapped cells and gray arrows indicated cells where WFA/ACAN positive signal colocalizes. Scale bar 100 μm. (**B**–**D**) Quantitative analysis of WFA positive or ACAN positive PNNs. Data showed mean ± SEM (*n* = 3 animals per groups, 3 sections per animal). *p < 0.05, **p < 0.01, ***p < 0.001, ****p < 0.0001, two-way ANOVA, Dunnett's multiple comparison. (**E**) Representative confocal images showing WFA positive and ACAN positive PNNs surrounding ChAT positive motoneurons in the thoracic rat spinal cord (Th8) in placebo, 4-MU treated and wash-out group in ventral horn. Scale bar 30 μm.

We next investigated the effect of 4-MU treatment (at a dose of 1.2 g/kg/day) on PNNs in the ventral horns by co-staining of WFA and ACAN. WFA is a widely used PNN marker and has been shown to specifically label the N-acetyl-D-galactosamine residue at terminal ends of chondroitin sulfate chains^32,33^. ACAN is a major PNN component and has been reported to be superior in labelling PNN positive motoneurons in the spinal cord^17^. Spinal cord sections from all 5 groups were stained for WFA (Fig. 3A, green arrows) and ACAN (Fig. 3A, red arrows), the number of positive cells in the ventral horns up to the central canal was counted (Fig. 3B–D). Similar to the biochemical assays, 4-MU treatment and treadmill exercise independently reduced the total number of WFA-positive cells in spinal ventral horns (Fig. 3B–D). The combination of both induced a stronger down-regulation, however, this did not reach significance. In addition, we also observed that the number of WFA and ACAN positive neurons returned to control levels after wash-out period. PNNs have been previously shown to enwrap α-motoneurons in the spinal cord. With the use of anti-ChAT antibody, we observed reduced PNNs around ChAT-positive neurons in thoracic uninjured spinal cords after 4-MU treatment (Fig. 3E). This suggests that the current 4-MU dose at 1.2 g/kg/day or rehabilitation can effectively reduce PNNs in the uninjured spinal cord.

### 4-MU (1.2 g/kg/day) is insufficient to down-regulate the increasing production of chondroitin sulfates after SCI

Having found that a 4-MU dose of 1.2 g/kg/day combined with rehabilitation could effectively reduce PNNs in the spinal cord, we next investigated whether this dose is sufficient to reduce the increased expression of inhibitory ECM in injured spinal cord. Rats received 200 kdyn impact to the Th8 spinal level which induced moderate SCI. Six weeks after the injury, the animals were divided into two groups, and the groups were fed a daily diet containing 4-MU- or placebo for 8 weeks. In addition, both groups received task-specific rehabilitation for the 16 weeks concurrent to the oral 4-MU treatment (i.e. 8 weeks during 4-MU treatment and 8 weeks afterwards) in order to prime appropriate re-connection from the potentially heightened neuroplasticity (Fig. 1, SCI).

**Figure 4:**
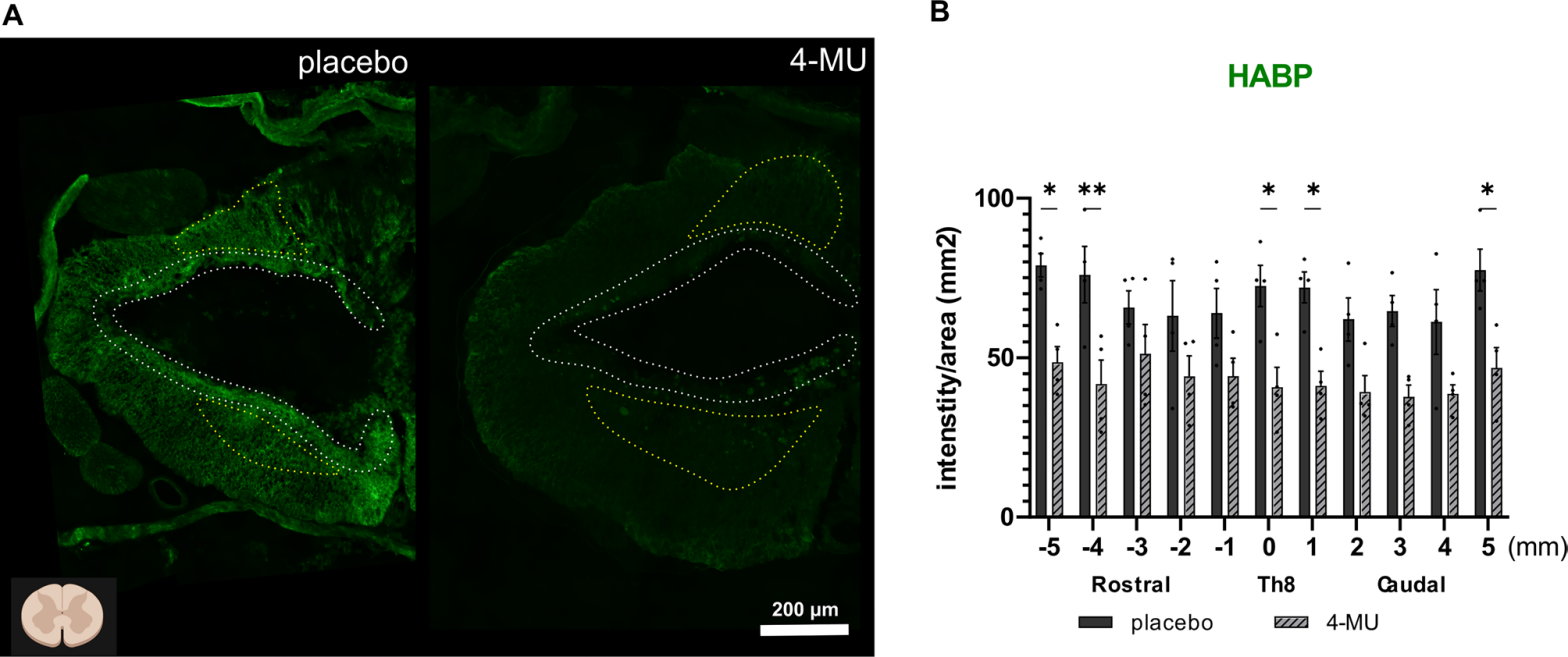
Hyaluronan binding protein (HABP) intensity remained decreased following chronic spinal cord injury, 8 weeks of 4-MU treatment and 8 weeks wash-out period combined with daily rehabilitation. (**A**) Representative fluorescent images showing different HABP positive signal intensity in spinal cord injured (SCI) rats in placebo and 4-MU-treated groups after 8 weeks of feeding and after 8 weeks of wash-out period. Bar graph shows the intensity per sections throughout the spinal cord. White dotted lines delineating the border of the cavity. Yellow dotted lines depicting the spared gray matter. The area of the spared gray matter differs among animals due to injury variability and individual character of the animals. 3 animals were excluded based on BBB test 1 week after the injury (i.e., animals with less-than 8 score in BBB test were not included in the present study). Diagram in the bottom left shows the orientation of the cross sections, created with BioRender.com. Scale bar 200 µm; (**B**) Quantification of (A). Individual data together with their mean ± SEM were shown (*n* = 3 animals per group). *p < 0.05, **p < 0.01, by two-way ANOVA, Sidak's multiple comparisons test.

First, we evaluated the level of HA within the spinal cord (Fig. 4) using HABP. There is a significant reduction of HA surrounding the lesion, rostrally (up to 5 mm) and caudally (1 and 5 mm) from the lesion in the 4-MU-treated group (40.78 ± 6.30) compared to placebo group (72.46 ± 6.46). We observed the trend of lower level of HA throughout the spinal cord after 4-MU treatment. The intensity remained decreased even after 8 weeks of wash-out period.

**Figure 5:**
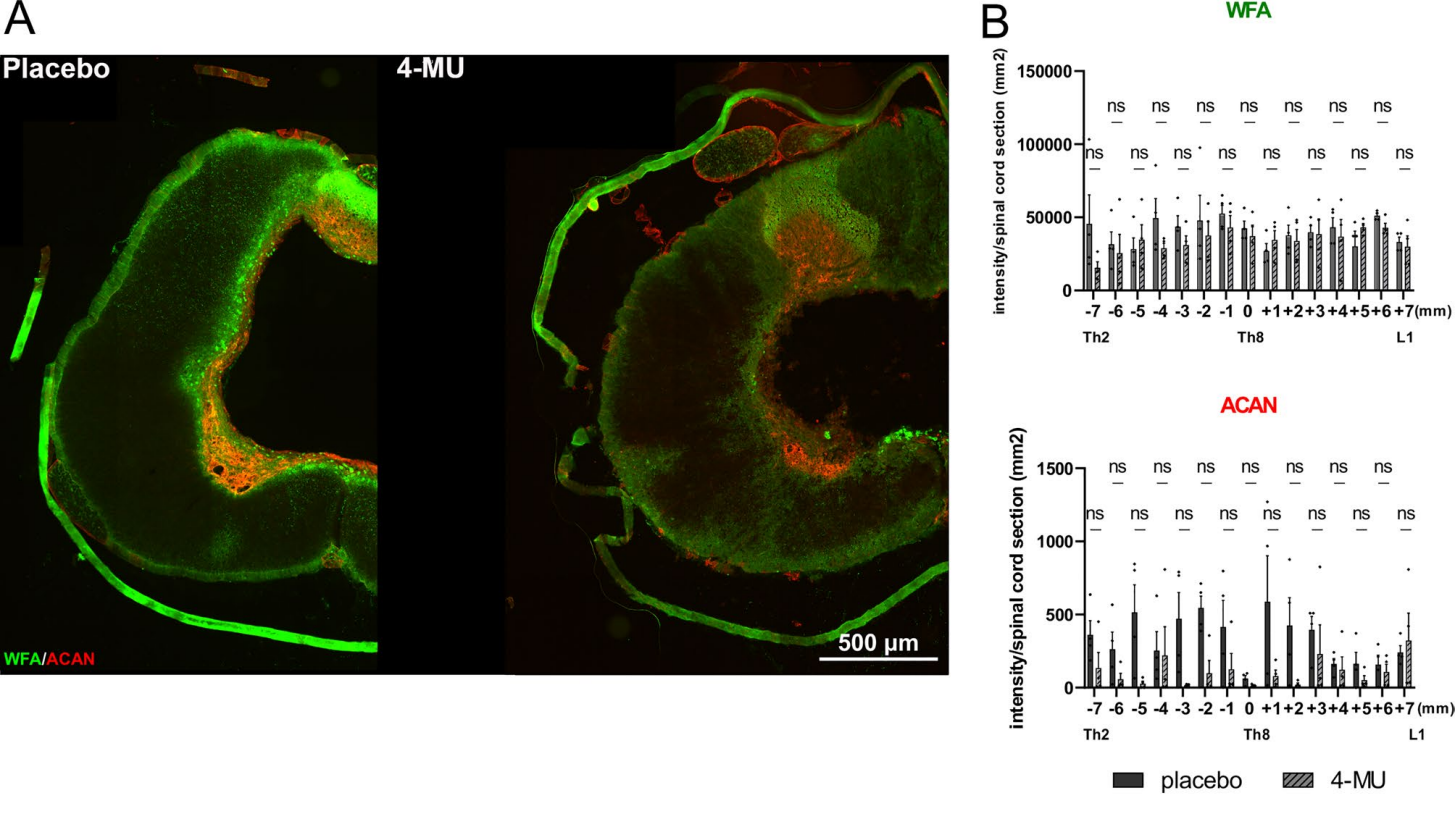
Immunofluorescence double-staining of *Wisteria floribunda* agglutinin (WFA) and aggrecan (ACAN) suggested that 4-MU at current dose 1.2 g/kg/day is not sufficient to down-regulate the increasing production of chondroitin sulfates after spinal cord injury (SCI). (**A**) Representative fluorescent images showing WFA positive (in green) and ACAN positive (in red) area around the centre of the lesion. Scale bar 500 μm; (**B**) the quantitative analysis of WFA or ACAN intensity. Bar graphs show the intensity per sections throughout the spinal cord with the lesion centre marked as level 0. Individual data points and their mean ± SEM were shown (*n* = 4 animals per group). ns by two-way ANOVA, Sidak's multiple comparisons test.

We then evaluated the level of PNNs and CSPGs using WFA and ACAN. Our results showed no significant difference between 4-MU-treated and placebo groups for 8 more weeks without any treatment but daily rehabilitation (Fig. 5). These findings correspond with our data from uninjured animals where PNNs re-appeared after 2 months wash-out period (Figs. 1 and 2). The strong up-regulation of CSPGs after SCI has rendered the dose of 1.2 g/kg/day 4-MU to be insufficient to supress their production in injured spinal cord.

### 4-MU reduces glial scar in chronic spinal cord injury

**Figure 6:**
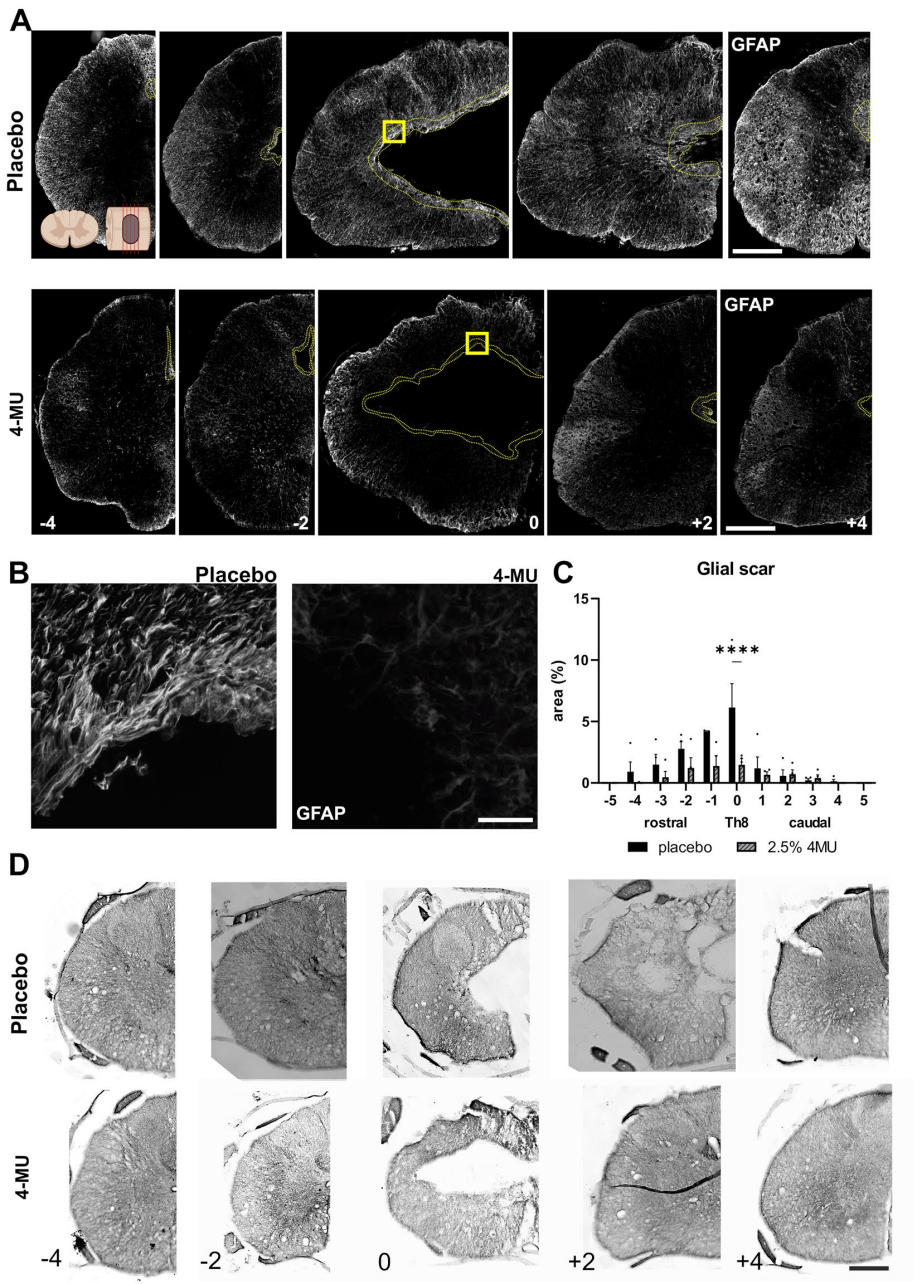
4-MU treatment reduced glial scar area surrounding the lesion site. (**A**) Representative fluorescent images showing lesion epicentre (0 mm), above (− 4, − 2 mm) and below (+ 2, + 4 mm) the lesion, stained for glial fibrillary acidic protein (GFAP) in placebo and 4-MU treated group with chronic spinal cord injury. Dotted lines show the border area of the lesion cavity in 4-MU treated group and GFAP positive area in placebo group. Scale bar: 200 µm. Diagram of uninjured spinal cord at top left showing the direction of the cross section in (**A**), created with BioRender.com; (**B**) magnified images (yellow square in **A**) showing structural change of the glial scar tissue after 4-MU treatment compared to placebo treated animals. Scale bar 30 µm; (**C**) bar graph showing area of the glial scar around the central cavity performed in the GFAP stained histochemical images using ImageJ software. Values are plotted as mean ± SEM; ****p < 0.0001 by two-way ANOVA, Sidak *post-hoc* test. (*n* = 4 animals per group). (**D**) Representative images of Luxol Fast Blue staining showing the lesion extension in a rostro-caudal direction. Scale bar 200 µm.

HA is produced by both neurons and glia in the CNS, and is up-regulated by astrocytes in neuroinflammation^34–36^. It has been previously suggested that 4-MU reduces astrogliosis in the brain parenchyma in the mouse model of the experimental autoimmune encephalomyelitis^37^. In view of our observation in HA down-regulation around the lesion cavity, we next focused on the effect of 4-MU to the glial scar after SCI. Quantitative analysis of GFAP-positive area was performed to assess the glial scar surrounding the lesion cavity on cross sections (Fig. 6). A significant decrease in astrogliosis was observed in 4-MU treated group at a dose of 1.2 g/kg/day compared to placebo group. The average peak in the centre of the lesion in 4-MU groups was 1.49 ± 0.51% (*n* = 4) and 6.1 ± 1.94% (*n* = 4) in placebo groups (Fig. 6C). It is also noted that the GFAP staining at the periphery of the 4-MU sections seem to be brighter than the placebo control groups. We thus analysed the pixel intensity between the two groups and did not observe any significant difference.

**Figure 7:**
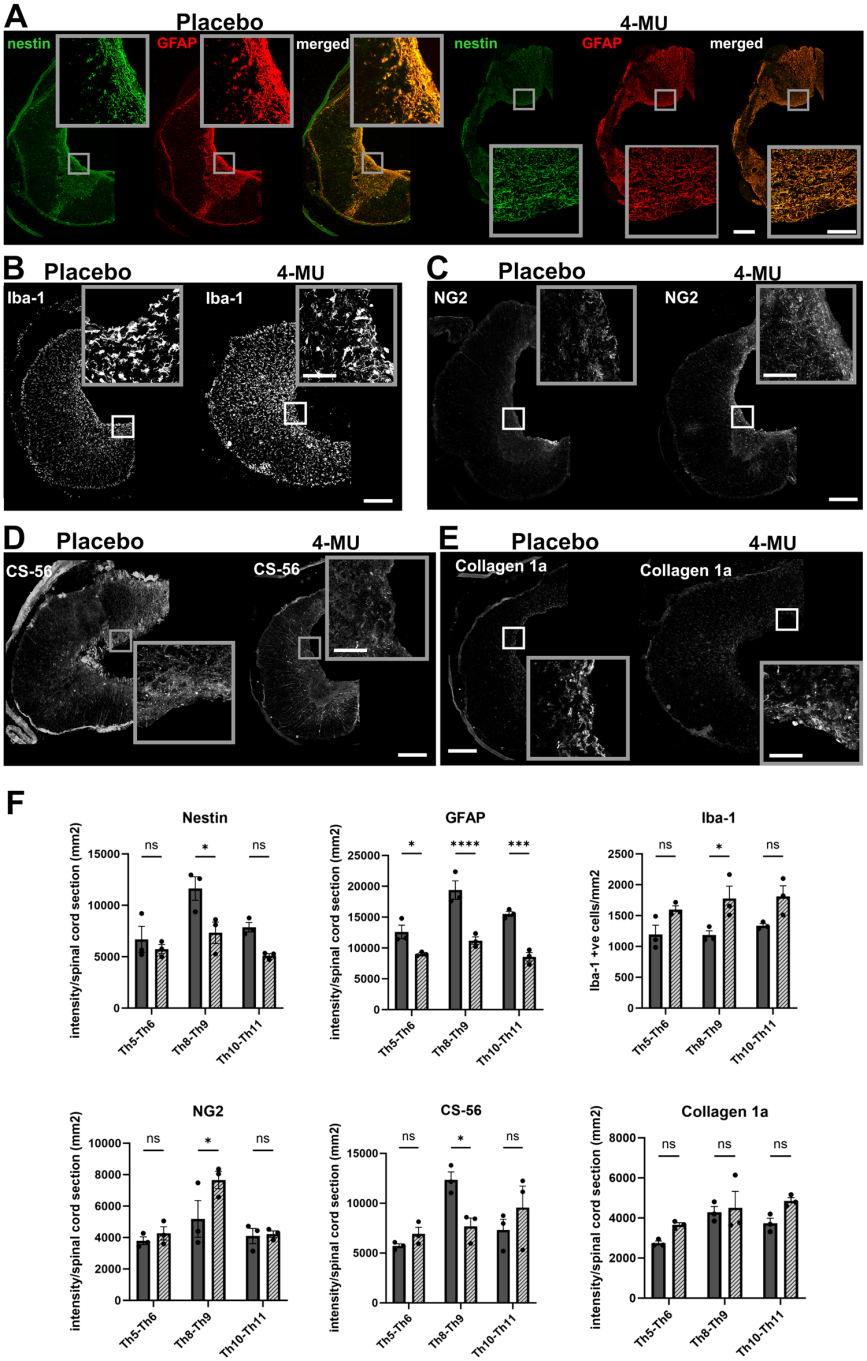
4-MU treatment leads to changes of cell and ECM composition around the lesion scar (Th8-9), above (Th5-6) and below (Th10-11) lesion. (**A**–**E**) Representative confocal images showing the 4-MU-mediated effect on scar-forming cells and components using different markers—(**A**) nestin and GFAP were used to visualise scar-forming astrocytes. (**B**) Iba-1 to visualise microglia/macrophages. (**C**) NG2 to visualise oligodendrocyte progenitor cells (OPCs). (**D**) CS-56 to examine the changes in CS sulfations. (**E**) Collagen 1a to visualise meninges and fibroblasts. All insets show magnified views of the staining. Scale bar 200 µm for the overview image and 50 µm for the insets. (**F**) Quantification of (**A**–**E**). Bar graphs show intensities per section throughout the spinal cord, except for Iba-1 staining where the number of Iba-1 positive cells per mm was counted. Individual data are shown with their mean ± SEM (n = 3 animals per group). p < 0.05, **p < 0.01, ***p < 0.001, ****p < 0.0001, by two-way ANOVA, Sidak's multiple comparison test.

To determine whether 4-MU affects only GFAP expression in scar-forming astrocytes or the astrocytes themselves, we performed immunohistochemistry for nestin and compared their behaviour to GFAP-positive cells around the lesion epicentre (Th8–Th9), below (Th10–Th11) and above (Th5–Th6) the lesion (Fig. 7F). Intensity measurement showed a reduction in signal for both nestin and GFAP after 4-MU treatment, when compared to placebo. This suggests that there is a reduction in astrocyte proliferation and activation.

We next checked how 4-MU treatment affects microglia/macrophages and oligodendrocyte progenitor cells (OPCs). We used the ionised calcium-binding adaptor molecule 1 (Iba-1) as a microglia/macrophage-specific calcium-binding protein (Fig. 7B,F). We observed a significantly higher number of Iba-1 positive cells at the lesion site after 4-MU when compared to the placebo group. This suggests that 4-MU mediates the infiltration of microglia and macrophages in the area.

Next, we examined the changes in OPCs using neuron-glial antigen 2 (NG2) and observed a significant increase in NG2 signal after long-term treatment with 4-MU followed by a 2-month washout period (Fig. 7C,F). As NG2-expressing OPCs can either be (i) contributing to scar-formation in response to the secretion of bone morphogenic protein (BMP) by the activated astrocytes or (ii) self-renewal and facilitates regeneration, further experiments will be required to confirm their functions at the lesion area. Nonetheless, these results demonstrated that 4-MU treatment is modulating the cellular composition around the lesion area.

To investigate the changes in the extracellular matrix around the lesioned area, we have examined the level of CSPGs and collagen 1a (Fig. 7D–F). With the use of CS-56 antibody which recognises CS-GAG chains, we observed a significantly down-regulation of CS-56 signal at the lesion epicentre in the 4-MU treated group when compared to the placebo group. This is in contrast to the WFA and aggrecan staining in Fig. 5 where no significant change was observed. As CS-56 is specific for chondroitin sulfate types A and C enriched sequence^38^, our results suggests that there is a differential regulation in sulfation pattern around the lesion site after 4-MU treatment. The mechanism of how 4-MU induces such changes is unclear.

We then examined the levels of collagen 1a which is produced by meninges or fibroblasts. There was no significant change in the presence of collagen 1a-positive cells at the lesion site or away from the lesion.

### Daily 4-MU at a dose of 1.2 g/kg/day promotes sprouting of serotonergic fibres distant from the injury but has no effect on synaptic density around the lesion site

**Figure 8:**
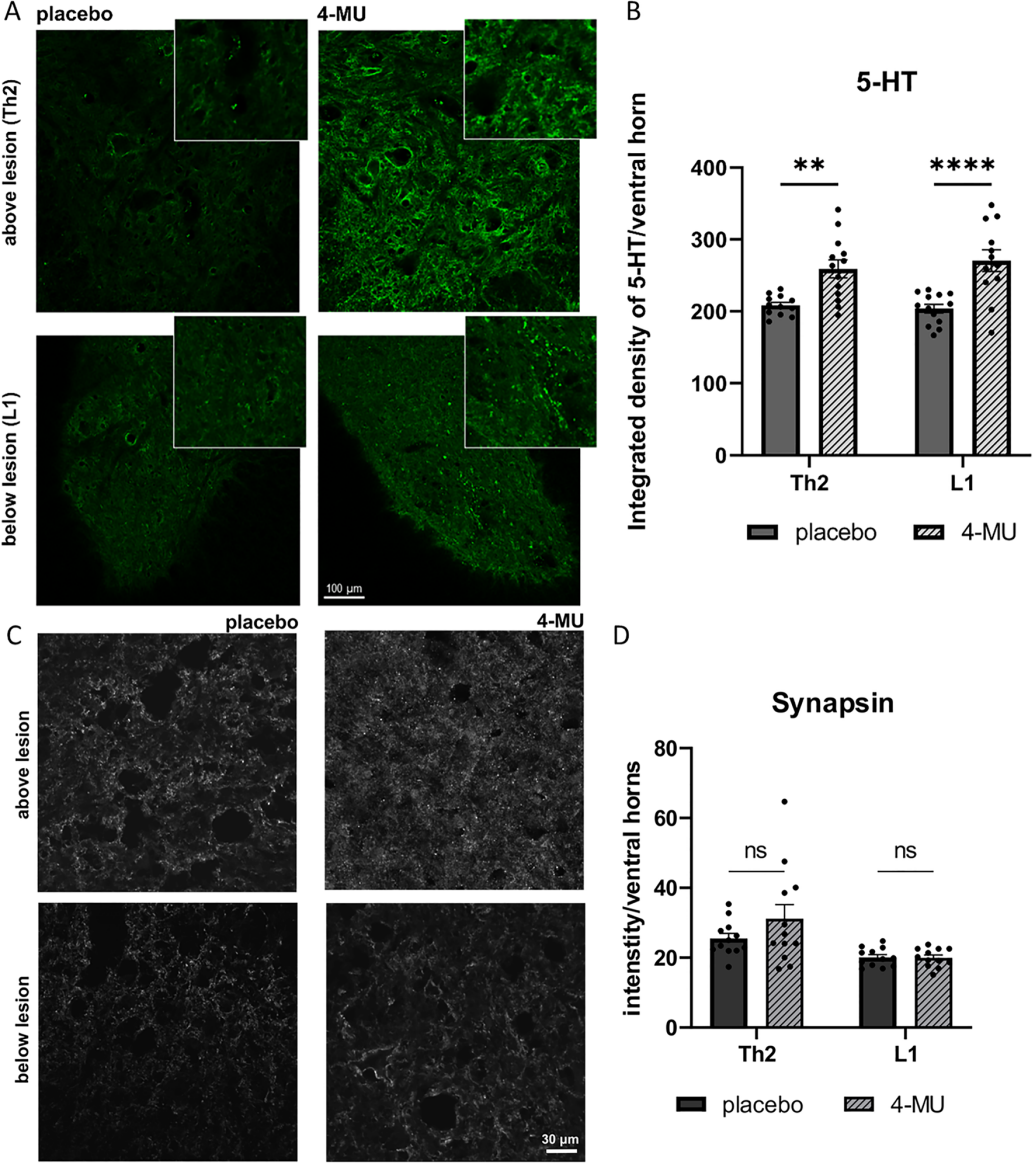
4-MU treatment increases the puncta of serotonergic fibre distant from the lesion but does not increase synapse number at the lesion site after chronic spinal cord injury (SCI). (**A**) Representative confocal images showing ventral horn above and below lesion stained for 5-hydroxytryptamine (5-HT) in placebo and 4-MU treated group in chronic stage of SCI. Insets show the magnified views of the staining. Scale bar 100 µm; (**B**) bar graph showing the integrated density of 5-HT positive signal in ventral horns using ImageJ™ software. Values are plotted as mean ± SEM (*n* = 4 animals per group; 2–3 sections per animal); **p < 0.01, ****p < 0.0001 by two-way ANOVA, Sidak's multiple comparisons test. (**C**) Representative confocal detailed images showing ventral horn above and below lesion stained for synapsin in 4-MU treated and placebo group in chronic stage of spinal cord injury. Scale bar 30 μm; (**D**) Bar graph showing intensity measurement per area (in pixels) of ventral horns using ImageJ software. Values are plotted as mean ± SEM (*n* = 4 animals per group; 3 sections per animal); ns > 0.05 by two-way ANOVA, Sidak's multiple comparisons test.

4-MU has previously been shown to down-regulate PNNs in the brain at a dose of 2.4 g/kg/day^24^. The results in Figs. 4, 5, 6 and 7 showed that while lower dose of 1.2 g/kg/day is able to reduce HA and glial scar, the level of CSPGs remains similar to untreated injured controls after SCI (Fig. 5), and that there are sulfation modifications on the CS-GAG chains. Serotonin (5-hydroxytryptamine; 5- HT) is an important neurotransmitter in the mammalian spinal cord that plays an essential role in controlling sensorimotor functions. We thus investigated if 4-MU treatment leads to any changes in serotonergic innervation. We observed that after an 8-week treatment with 4- MU and a 2-month washout period plus rehabilitation, there was a significant increase in 5-HT positive puncta in the ventral horns above the lesion in the 4- MU treated group (259.25 ± 12.3) compared to the placebo group (208.58 ± 4.06), and below the lesion in the 4-MU treated group (270.67 ± 15.17) compared to the placebo group (203.96 ± 6.01) (Fig. 8A,B). The results suggest that 4-MU treatment, in combination of rehabilitation during the 8-week washout period, leads to the downregulation of HA and promotes long-term synaptic plasticity after chronic spinal cord injury.

To investigate whether there is a difference in overall synaptic density (Fig. 8C,D), we stained 2 levels above and below the lesion for the presynaptic marker synapsin and measured the level of synaptic contacts within ventral horns. 4-MU treated animals showed a trend of increased synaptic density in the area above lesion, but with no significant difference (Fig. 8D). There is no change in synaptic density measurement below lesion, probably due to the lack of CS reduction as observed in Fig. 5.

### 4-MU at a dose of 1.2 g/kg/day is not sufficient to enhance inherent functional recovery in chronic stage of spinal cord injury

**Figure 9:**
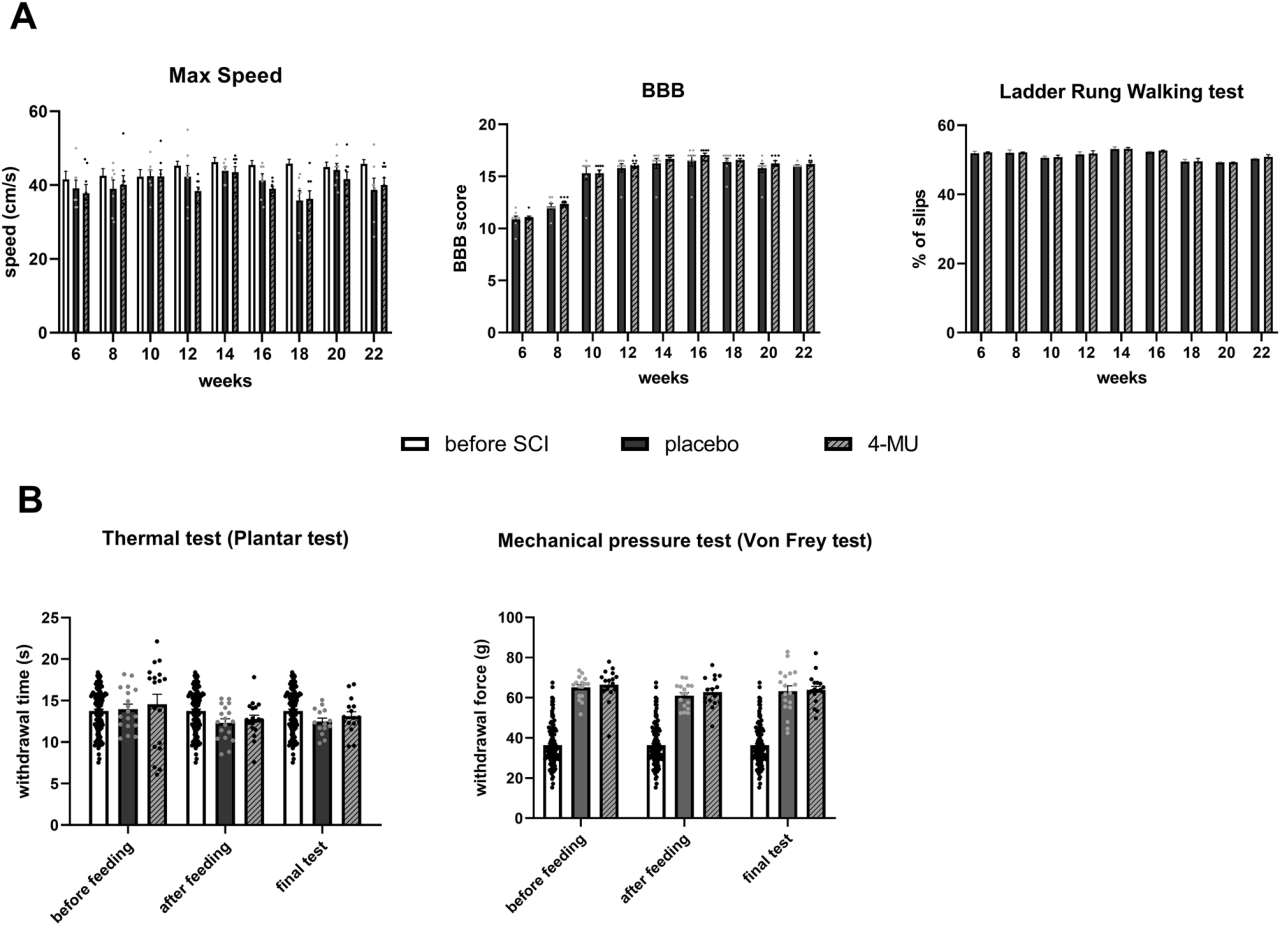
4-MU treatment at a current dose does not lead to functional recovery after chronic SCI. The animals were tested weekly for their ability to reach the highest possible speed on the treadmill (max speed), to move around in open-field test (Basso, Beattie, Bresnahan—BBB), dexterity (ladder rung walking test), and sensory functions (Plantar and von Frey tests) at the three different time points of the experiment. (**A**) The bar graphs show the results of the behavioural tests assessing locomotion in rats—maximum speed (max speed), BBB, ladder rung walking test. The data show spontaneous recovery in both groups after the first two weeks of daily rehabilitation in the BBB Open Field Test, which stagnated in both groups at week 10. There was no 4-MU-mediated enhancement of this spontaneous recovery. Values are presented as mean ± SEM (*n* = 7 animals for the placebo group and *n* = 8 animals for the 4 MU group); ns > 0.05 by two-way ANOVA, Sidak's multiple comparison test (BBB, ladder rung walking test); ns > 0.05 by two-way ANOVA, Tukey's multiple comparison test (max speed). (**B**) The bar graphs show sensory function scores—before and after feeding and at the end of the experiment. No significant difference was found. Values are presented as mean ± SEM (*n* = 7 animals for the placebo group and *n* = 8 animals for the 4-MU group); ns > 0.05 by two-way ANOVA, Tukey's multiple comparison test.

On account of biochemical results showing that 4-MU abolishes plasticity-limiting perineuronal nets, we tested if the axonal sprouting induced by 4-MU and daily rehabilitation would lead to functional recovery in chronic stage of SCI. To test this, the whole battery of behavioral tests was performed, including weekly BBB test, maximum speed test and ladder rung walking test combined with daily rehabilitation on treadmill assessing their locomotor abilities. Two sensory tests, mechanical pressure test (Von Frey test) and thermal test (Plantar test) were chosen to assess changes in thermal and mechanical sensation. These two tests were performed trice—before feeding with 4-MU-containing/placebo pellets, at the end of feeding period, and at the end of the whole experiment. In our results, there were no significant differences or indication for a trend of improvement at any time point suggesting the lack of 4-MU-mediated recovery (Fig. 9).

## Discussion

We investigated whether a dose (1.2 g/kg/day) of 4-MU was sufficient to decrease PNNs in ventral horns, and to promote sprouting and functional recovery in chronic SCI. Previous study in mice using 2.4 g/kg/day has led to an enhancement neuroplasticity for memory acquisition through PNN down-regulation^24^. As the LD_50_ is slightly lower in rats than in mice, we therefore tested whether reducing this dose to half, i.e., 1.2 g/kg/day, would be able to down-regulate PNNs for possible functional recovery after SCI in rats. We found that the orally administered dose of 1.2 g/kg/day (4-MU) was sufficient to reduce PNNs and HA in uninjured animals, however, this is not sufficient to suppress the strong CSPGs upregulation after SCI, so that functional recovery was not observed.

In recent years, many studies have shown strategies focusing on regeneration after SCI, often based on targeting PNNs and manipulating the glial scar to attenuate inhibitory properties of its environment. Current strategies range from proteolytic ECM manipulation to targeting the specific ECM components through the synthesis of inhibitory ECM molecules after SCI^39^. One of the most studied approaches has been an enzymatic ECM modification using ChABC. ChABC degrades CS chains into disaccharides and removes CSPG inhibition in the glial scar as well as removes the PNNs as plasticity brake, to benefit both acute and chronic SCI conditions^40^. Animals with SCI show better recovery both anatomically and functionally after ChABC treatment^19,20,40^. Functional recovery after SCI is further boosted when plasticity restoration is combined with rehabilitation^20,40^. However, ChABC application has several disadvantages. The major disadvantage of using this bacterial enzyme is its thermal instability and short half-life, which requires multiple or continuous intrathecal administrations^41^, potential immune response of the body and difficult dosing. Apart from ChABC, Keough and colleagues^1^ were testing a subset of 245 drugs known for their CNS penetrating capacity and oral bioavailability. None of these 245 compounds showed sufficient ability to overcome CSPG inhibition from oligodendrocyte precursor cells.

4-MU, the derivative of coumarin, used for biliary stasis therapy, is a HA inhibitor^25^. It has been previously shown to reduce the synthesis of HA by decreasing the production of UDP-GlcA, a key monosaccharide substrate for the synthesis of HA^42^, and the expression of HASs. As UDP-GlcA is also a substrate for CS production, we thus investigate if 4-MU administration would decrease the synthesis of both HA and CS, facilitating neuroplasticity. Indeed, we have observed the down-regulation of HA and CS, both anatomically using histochemistry and biochemically through GAG quantification (Fig. 2). Our data suggest that 4-MU combined with daily training suppresses the synthesis of GAG. With the use of PNN markers including WFA and ACAN, 4-MU administration led to PNNs removal in ventral horns. PNNs re-appeared after 2 months of wash-out period. The reason why PNNs reappeared while the HA level remained low after 2 months of wash-out is likely because CS synthesis is less sensitive to the UDP-GlcA deficiency. CS is synthesized in the Golgi apparatus where UDP-GlcA sugars are being transported to the Golgi lumen with high affinity, while HA is synthesized directly at the cytoplasmic membrane^25^.

We used a thoracic contusion injury that spares some axons around a central cavity and ablates dorsal corticospinal tracts (CSTs) critical for motor control in humans, thus mimics the type of closed SCI most commonly seen in humans^43^. The 4-MU treatment started in the chronic stage, *i.e.* 6 weeks after the injury^44^. At this stage, glial scar is well-established, CSPGs are upregulated, and the acute immune response had already subsided^45,46^. 4-MU treatment was accompanied by daily rehabilitation on treadmill to consolidate appropriate synaptic connections and prune away others^47^. After the 8 weeks of treatment and rehabilitation, rehabilitation was continued for another 8 weeks for the wash-out. This wash-out period gives time for PNNs to re-form and stabilize de novo synapses and consolidate anatomical plasticity^20,22,40^, while the continuing rehabilitation prunes random connections, supporting appropriate connections and removing inappropriate ones^48,49^. Oral administration of 1.2 g/kg/day 4-MU robustly decreased the glial scar surrounding the cavity that persisted throughout the wash-out period.

To evaluate the potential of 4-MU to remove the plasticity brake formed by PNNs, we analysed the intensity of 5-HT signal and observed increased 5-HT sprouting distant from the lesion. However, this sprouting did not lead to any significant difference in synapsin immunoreactivity within the ventral horns, above and below the lesion, between the 4-MU-treated and placebo animals. We also investigated the effect of 4-MU in mediating changes in other cellular composition. Indeed, we observed a reduction in both GFAP and nestin staining suggesting a reduction in astrogliosis. In addition, we observed an upregulation of Iba-1 and NG2 staining, suggesting mobilisation of microglial/macrophages and OPC into the area. It is not yet clear at this stage if their mobilisation is resulting from a direct effect of 4-MU treatment or the modulation of ECM resulting from 4-MU. Nonetheless, this suggests that 4-MU is modulating immune cell population in the lesioned environment. Assessment of functional recovery using the BBB test, maximum speed test and ladder rung walking test yielded no significant differences between the 4-MU and placebo treated groups, even with the continuous rehabilitation for next 2 months. As 4-MU-mediated PNNs ablation has been already demonstrated in previous work focused on mouse hippocampus where it enhances memory in ageing mice^24^, we reasoned that the lack of functional recovery after SCI in this study is due to the lower dose of 4-MU administered (1.2 g/kg/day *versus* 2.4 g/kg/day) and the strong CS-GAG upregulation after injury. A higher dose of 4-MU combined with rehabilitation should be tried for its effect on recovery after SCI.

The promising results we observed in the 5-HT staining are consistent with the published data. Previous studies have already shown that severe SCI paralysis is caused not only by a loss of direct muscle innervation by spinal motor neurons, but also by a loss in the supraspinal pathways involved in voluntary initiation of movements and by a loss in the descending pathways that supply motor neurons with neuromodulators such as 5-HT^50,51^. In the intact spinal cord, serotonergic innervation emanates almost entirely from the raphe nuclei in the brainstem, which are lost^52,53^ or severely disrupted^54^ after SCI. The drastic and abrupt decrease in serotonin availability below the lesion results in spinal networks that are no longer excitable or responsive^55,56^, contributing substantially to SCI -induced paralysis^53^. Barzilay and colleagues have also shown that HA is involved in the propagation of serotonergic fibres thereby improving neuroplasticity^57^. 5-HT stimulates spinal interneurons and motoneurons, allowing appropriate muscle contraction^50,58^. In acute SCI, spinal motoneurons and interneurons lack 5-HT^59,60^ resulting in paralysis and spinal shock^61,62^. Therefore, in chronic SCI, motor neurons partially recover their excitability despite the continued absence of 5-HT^63^. It has also been suggested that this compensatory mechanism requires activity-dependent tuning to contribute to motor activity following SCI^52^. Our behavioural data have also shown spontaneous recovery of locomotor activity in rats with the contusion model of SCI. This spontaneous recovery is not only important in animals, but also in human patients with incomplete SCI. In incomplete SCI, part of the descending connection to the brain is spared, so animals, as well as human patients, can learn to use these spared connections to achieve substantial recovery of walking function, especially through rehabilitative training^64–66^. Understanding and promoting this plasticity in spared connections is therefore an important focus of spinal cord research. For example, it is already known that spared corticospinal axons sprout above the injury and form new connections, including transmission of descending inputs around the injury through spared propriospinal pathways^67–69^. In addition, spared descending fibres sprout below the injury, a process that has been well correlated with recovery in animal models^66^. For these spared and new connections to be functional, neurons downstream of the injury must be ready to respond to these enhanced or restored descending signals. This probably requires compensation for the loss of neuromodulatory (5-HT) innervation. In vitro time-lapse imaging studies have shown that serotonergic growth cones are more active than cortical growth cones when challenged with CSPG, and that serotonergic neurons recover better after enzymatic digestion with ChABC^70^. These results suggest that 4-MU may cause serotonergic fibres to respond better to treatment in vivo as well, through its effect on CSPG expression. In the case of ChABC, the studies have shown that this could be at least partly due to differences in the cytoskeleton or receptors. We speculate that systemic administration of 4-MU together with overall rehabilitation on treadmill induced 5-HT sprouting in the spinal cord even though CSPGs were not downregulated around the lesion. The robust response of serotonergic neurons may also be related to increased expression of GAP-43, which is associated with axon growth or regeneration and is normally downregulated in adulthood^71,72^. Serotonergic neurons require GAP -43 postnatally for proper terminal arborisation, at least in the forebrain^73^. Moreover, serotonergic neurons also produce GAP -43 mRNA in adulthood^74^, possibly increasing the sensitivity of 5-HT fibres to injury.

In order to determine if there is any compensatory mechanism for the loss of HA synthesis, we have evaluated the changes in mRNA expression for genes related to the HA synthesis (*Has1*, *Has2*, *Has3*)^25^ (Fig. 2D). We did not observe any significant difference despite the clear trend of down-regulation in their mRNA levels. The *has* genes expression after 1 mM 4-MU treatment, studied on human aortic smooth muscle cells showed reduction mainly in *Has1* and *Has2* transcripts after 4-MU treatment^75^. To determine the biochemical effect of 4-MU at 1.2 g/kg/day on the HA synthesis in uninjured animals, we used histochemical staining for recombinant HABP derived from human versican G1 domain which binds specifically to hyaluronan and does not bind to other glycosaminoglycans (Amsbio; data sheet). We observed that after 2 months of wash-out period, the level of HA remains decreased compared to healthy animals. This may pose a question of whether the HA down-regulation after 2 months of wash-out period may induce adverse effects after treatment.

Clinical trials with 4-MU (drug approved in Europe and Asia as hymecromone) showed excellent safety parameters during long-term administration of approved doses—1.2–3.6 g/day^76–82^. The longest reported duration of oral 4-MU administration was for 3 months in human patients^80^ which is much shorter time according to correlation between age of laboratory rats and humans^83^ and it might be taken into consideration while explaining these results as the safety profile of long-term 4-MU treatment is not fully established yet^25^. However, our recent study in healthy rats showed that long term oral application of 4-MU at a dose of 1.2 g/kg/day did not result in any serious adverse effects. When deviations from reference levels occurred, they normalised after a 9-week wash-out period in Wistar rats. Our results suggest that 4MU at a dose of 1.2 g/kg/day is suitable for long-term treatment^30^.

In conclusion, this study demonstrates that oral administration of 1.2 g/kg/day of 4-MU reduces the total amount of GAGs, reduces glial scar and increases 5-HT puncta in chronic stage of SCI. However, these structural changes do not result in increased synaptic connections and are insufficient to induce functional recovery even after intensive rehabilitation. A higher dose of 4-MU is likely to be necessary to induce the functional recovery supported by synaptic plasticity.

## Methods

All methods were performed in accordance with the relevant guidelines and regulations. All procedures on animals were approved by the ethical committee of the Institute of Experimental medicine of Academy of Science of the Czech Republic (ASCR) and performed in accordance with Law No. 77/2004 of the Czech Republic (Ethics approval number: 13/2020). Power calculation, based on previous studies, was performed prior to the experiment to estimate the number of animals needed. All work was performed according European Commission Directive 2010/63/EU, and the ARRIVE guidelines. Efforts were made to minimize pain and suffering.

### Experimental animals

55 female Wistar RjHan:WI rats (8-week-old, 250–300 g; 2020–2021; CS 4105 Le Genest Saint Isle; Saint Berthevin Cedex 53941 France) were used in total. 40 animals were used for the histochemical and biochemical assessment of the 1.2 g/kg/day 4-MU dose effect in 4-MU-fed/non-fed combined with/without rehabilitation groups. This dose was chosen as we aim to test if a lower dose at 1.2 g/kg/day weight will be effective in reducing inhibitory ECM and at the same time, minimize the 4-MU consumption for potential adverse effects of long-term treatment for SCI^30^. These animals were divided into 5 groups (8 rats per group) using simple randomization—placebo, placebo + training/rehabilitation, 4-MU, 4-MU + rehabilitation, and 4-MU with planned 2 months post-feeding period (wash-out group). Based on our previous experiments, we are expecting an effect size of ≥ 1.7. A power calculation of α = 0.05, number of groups = 5, a total sample size of 8 is required for each experimental group.

Hereafter, the histochemical and biochemical assessment of the treatment in uninjured animals was done, 18 animals underwent the spinal cord contusion, 3 animals with BBB score less than 8, one week after the injury were excluded. The feeding has begun 6 weeks after SCI when the chronic phase was fully established. Half of the animals received chocolate-flavoured chow containing 4-MU (1.2 g/kg/day) (treated group) and the second half just chocolate-flavoured chow without any treatment (placebo group). Placebo and treated groups received daily physical rehabilitation on treadmill. Feeding stopped after 8 weeks, but daily extensive rehabilitation continued for 2 more months.

Rats were housed by two in cages with 12 h light/dark with standard conditions (in temperature (22 ± 2 °C) and humidity (50% ± 5%)). Rats had free access to water and food ad libitum. All experiments were performed between 08:30 and 19:00 local time.

### Animal surgeries

Animals received a moderate thoracic spinal cord contusion using a commercially available Infinite Horizon (IH) spinal cord injury device (IH-0400 Spinal Cord Impactor device; Precision Systems and Instrumentation, Lexington, KY, USA). Rats were anesthetized with 5% (v/v) isoflurane and maintained at 1.8–2.2% during the surgery, in 0.3 L/min oxygen and 0.6 L/min air. Animals were injected subcutaneously with buprenorphine (Vetergesic® Multidose, 0.2 mg/kg body weight). Using sterile procedures, a laminectomy was performed at the Th8/Th9 level with the vertebral column being stabilized with Adson tissue forceps at Th7 and Th10. The animals received 200 kilodynes (kdyn) moderate contusion injury. Manual expression of bladders was performed in the first two weeks after injury, and the rats were checked daily. Rats were given treatment of analgesics if signs of inflammation were observed. Animals were given a random number after the surgeries. The chow or the placebo was similarly given a random number, and fed to the animals according to the number. The experimenters were blinded to the treatment group during the behavioural analysis. The identity of the animals and their treatment group was revealed only after the evaluation.

At the end of the experiments, half of rats from each group was intraperitoneally anesthetized with a lethal dose of ketamine (100 mg/kg) and xylazine (20 mg/kg), perfused intracardially with 4% (w/v) paraformaldehyde (PFA) in 1X-PBS and post-fixed in the same solution for 24 h. Spinal cords were dissected before storing in 30% (w/v) sucrose (Milipore, cat. no. 107651) in 1X PBS. The second half of the rats were sacrificed with a lethal dose of ketamine (100 mg/kg) and xylazine (20 mg/kg) before the spinal cords were dissected and froze on dry ice before storage at -80°C for subsequent qPCR and GAGs extraction. The anaesthesia used have previously been shown not to interfere with animal behaviour.

### 4-MU treatment

2.5% (w/w) 4-MU was mixed and prepared into rat chocolate-flavoured chow (Sniff GmbH, Germany). This percentage would allow the delivery of 4-MU at 1.2 g/kg/day to the rats when chow was consumed ad libitum, an amount assessed from our pilot experiment. Rats were fed with the 4-MU chow at 10 weeks of age (non-SCI cohort) or 6 weeks after the SCI (SCI-cohort). Rats were maintained on 4-MU diet for 8 weeks. The food intake was measured weekly to check for consumption (Fig. 1).

### Histological and histochemical analysis

Two cm long spinal cords, with the Th8 level in the middle, were embedded in O.C.T. compound (VWR) and sectioned in frozen block of tissue before being sectioned into 40 μm thick slices for immunohistological analysis. The sections were made in series of 1 mm, compared to the anatomical atlas, and thus the spinal cord segments were identified.

40 µm sections (free-floating for uninjured spinal cords, and mounted on glass slides for injured spinal cords) were permeabilizated with 0.5% (v/v) Triton X-100 in 1X PBS for 20 min and then endogenous biotin was blocked using Avidin/Biotin blocking kit (Abcam; cat. no. ab64212) to reduce non-specific background. Tissue was then blocked in ChemiBLOCKER (1:10; Millipore cat. no. 2170), 0.3 M glycine, 0.2% (v/v) Triton X-100 in 1X PBS for 2 h. The sections were then incubated with labelling agents including biotinylated *Wisteria floribunda* agglutinin (1:150, 24 h), biotinylated hyaluronan binding protein (HABP) (1:150, 24 h) and/or primary antibodies: anti-choline acetyltransferase (ChAT)(1:600, 72 h); anti-ACAN (1:150, 24 h), anti-GFAP conjugated to Cy3 (1:800, 24 h), anti-5-HT (1:400; 24 h) or anti-synapsin (1:100; 48 h), anti-nestin (1:300, 48 h), anti-NG2 (1:300, 48 h), anti-GFAP (1:300, 48 h), anti-CS-56 (1:100, 24 h), anti-IBA1 (1:300, 48 h), and anti-collagen 1a (1:200, 48 h) as shown in Table 1. After washing, the sections were labelled with fluorescent-conjugated secondary antibodies (1:300; 2 h; room temperature (RT) (Table 1). Staining was imaged with a LEICA CTR 6500 microscope with FAXS 4.2.6245.1020 (TissueGnostics, Vienna, AT) software. Images were evaluated for the total number of cells in ventral horns with WFA-positive signal, ACAN-positive signal and number of co-localised cells using ImageJ™ (NIH, Bethesda, MD, USA). The HABP intensity was analysed with HistoQuest 4.0.4.0154 (TissueGnostics) software. 5-HT was imaged with confocal microscope Zeiss LSM 880 and synapsin images were taken with confocal microscope Olympus FV10i (Olympus Life Science, Waltham, MA, USA). The intensity of 5-HT and synapsin was measured in the ventral horn by ImageJ™ and compared between 4-MU-treated and placebo group. To quantify Iba-1-positive, the Analyse Particles function in the FIJI software was used (22,743,772).

**Table 1:** A table showing the primary antibodies and fluorescent-conjugated secondary antibodies used for the experiments.

1° Antibody/labelling agent	Epitope	Host	Company
Biotinylated *Wisteria floribunda* agglutinin (WFA)	Chondroitin sulfate in the PNNs	N/A	Sigma-Aldrich cat. no. L1516
Biotinylated hyaluronan binding protein (b-HABP)	Hyaluronan	N/A	Amsbio cat. no. AMS.HKD-BC41
Anti-ACAN	Aggrecan	Rabbit polyclonal	Sigma-Aldrich cat. no. AB1031
Anti-GFAP-Cy3	Glial fibrillary acidic protein	Mouse monoclonal IgG	Sigma-Aldrich cat. no. C9205
Anti-ChAT	Choline acetyltransferase	Mouse monoclonal IgG	Invitrogen cat. no. MA5-31383
Anti-5-HT	5-hydroxytryptamine	Mouse monoclonal IgG	Invitrogen cat. no. MA5-12111
Anti-synapsin	Synapsin	Rabbit polyclonal	Novus biologicals cat. no. NB300-104
Anti-Nestin	Nestin	Mouse monoclonal IgG1	Chemicon cat. no. MAB353
Anti-NG2	Neural/glial antigen 2	Rabbit polyclonal	Chemicon cat. no. AB5320
Glial fibrillary acidic protein	Glial fibrillary acidic protein	Chicken polyclonal	Abcam cat. no. ab4674
Anti-CS-56	Chondroitin Sulfate	Mouse monoclonal IgM	Sigma-Aldrich cat. no. C8035
Anti-IBA1	Ionized calcium-binding adapter molecule 1	Rabbit polyclonal	FUJIFILM Wako Pure Chemical Corporation cat. no. 019-19741
Anti- Collagen 1a	Collagen, type I	Mouse monoclonal IgG1	Abcam cat. no. ab6308

2° antibody/labelling agent	Company		
Streptavidin-Alexa fluor 488	Invitrogen cat. no. S32354		
Goat anti mouse Alexa fluor 488	Invitrogen cat. no. A11029		
Goat anti rabbit Alexa fluor 594	Invitrogen cat. no. A11012		
Goat anti mouse Alexa fluor 405	Invitrogen cat. no. A48255		
Goat anti mouse Alexa fluor 488	Invitrogen cat. no. A21042		
Goat anti rabbit Alexa fluor 594	Invitrogen cat. no. A11012		

For images where intensity was measured and compared, experimental conditions including for immunohistochemistry and microscopy were kept the same. The stained sections were imaged with the same laser power, gain and airy units.

Luxol Fast Blue (LFB) staining was used to visualise white and grey matter. LFB staining is used to identify myelin in nerve tissue. Spinal cord tissue was sectioned in 4 animals per group. A total of fifteen sections, including the centre of the lesion and both cranial and caudal areas, were observed and captured using a Zeiss LSM 880 Airyscan (Zeiss, Oberkochen, Germany).

### GAGs extraction and analysis

Frozen tissues were first weighted before the incubation with acetone to remove the lipids. The samples were then dried and cut into small pieces before the pronase treatment (15 mg pronase per hemisphere, (Roche, cat. no. 11459643001) in 0.1 M Trizma hydrochloride (Sigma-Aldrich, cat. no. T3253), 10mM calcium acetate (Millipore, cat. no. 567418), pH 7.8. Samples were homogenized with Potter Elvehjem tissue homogenizer in the pronase solution. Residual protein fragments were precipitated with trichloroacetic acid (Sigma-Aldrich, cat. no. T6399). The supernatant, which contains the GAGs, was collected and stored on ice. The solution was then neutralised with 1 M Na_2_CO_3_ (Sigma-Aldrich, cat. no. S7795) to pH 7.0, and the GAGs were recovered by ethanol precipitation. The isolated GAGs were redissolved in 0.3 ml of deionised water. The GAG concentration was quantified using cetylpyridium chloride (CPC) turbidimetry. Standard curve was prepared from 1µg/µl of Chondroitin sulphate A (Sigma-Aldrich, cat. no. C9819)^31^. Briefly, the diluted sample was mixed with 0.2% (w/v) CPC and with 133 mM MgCl_2_ (Sigma-Aldrich, cat. no. M2670) in ratio 1:1. Absorbance was measured at 405 nm using plate reader spectrophotometer (FLUOstar® Omega, BMG LABTECH). Each sample was carried out in three different dilutions and each dilution was carried out in duplicate.

### Quantitative real-time polymerase chain reaction

For quantitative evaluation of gene transcript levels in spinal cords treated with or without 4-MU, quantitative real-time PCR (qPCR) was used. RNA was isolated with RNeasy© Lipid Tissue Mini Kit (QIAGEN, cat. no. 74804) according to the manufacturer’s protocol. Amount of isolated RNA was quantified using NanoPhotometer© P330 (Implen, München, Germany). Then, TATAA GrandScript cDNA Synthesis Kit (TATAA Biocenter, Art No. AS103c) was used for reverse transcription of RNA into complementary DNA (cDNA), following the manufacturer’s protocol in the T100TM Thermal Cycler (Bio-Rad, Hercules, CA, USA). For the qPCR, TaqMan® Gene Expression Assays (Life Technologies by Thermo Fisher Scientific, Waltham, MA, USA) were used for HAS1 (Rn01455687_g1), HAS2 (Rn00565774_m1), HAS3 (Rn01643950_m1) and GAPDH (Rn01775763_g1), all purchased from Applied Biosystems and used as recommended by the manufacturer. Amplification was performed on the qPCR cycler (QuantStudio™ 6 Flex Real-Time PCR System, Applied Biosystems® by Thermo Fischer Scientific, Waltham, MA, USA). All amplifications were run under the same cycling conditions: 2 min at 50 °C, 10 min at 95 °C, followed by 40 cycles of 15 s at 95 ◦C and 1 min at 60 ◦C. Expression was calculated using the threshold cycle (Ct) value and log(2^−ΔΔCt^) method and normalized to the control group. Each qPCR experiment was carried out in duplicate. Ct values of each measured condition were normalized to glyceraldehyde 3-phosphate dehydrogenase (GAPDH). Then, the 2^−ΔΔCt^ values were expressed as described by Livak and Schmittgen^84^. After that, the mean of each subset was calculated for each group.

### Behavioural tests

#### Treadmill training

Treadmill training began 6 weeks after SCI and was conducted on 5 consecutive days per week for 16 weeks. The training consisted of 10 min run, 20 min break and 10 min run. The treadmill speed was 16–18 cm/s in the first and second weeks and 20 cm/s in the remaining weeks of training. Half of non-SCI rats were trained for 8 weeks as well, except wash-out group when the training had been prolonged for 2 months without 4-MU feeding.

#### Maximum speed test

Maximum Speed test was performed once per week and began 6 weeks after the SCI. The treadmill speed started at 20 cm/s and was increased every 10 s for 2 cm/s. When the animal was not able to run at a given speed, the treadmill was stopped and the last value was recorded.

#### Basso, Beattie and Bresnahan (BBB) test

The Basso, Beattie and Bresnahan (BBB)^85^ open-field test was used to assess the locomotor ability of the rats. The rats were placed into the arena bordered with rectangular-shaped enclosing. The results were evaluated in the range of 0–21 point; from the complete lack of motor capability (0) to healthy rat-like locomotor ability (21). The measurements were performed 4th and 7th day after the SCI and then weekly for 8 weeks, starting 6 weeks after SCI.

#### Ladder rung walking test

For the advanced locomotor skills, the ladder rung walking test had been used. Animals were placed on a 1.2 m—long horizontal ladder, with irregularly spaced rungs. At the end of the ladder was a dark box that the rats favoured. The animals crossed the ladder 3 times in a row and all attempts were recorded on camera. The hind paws placement on the rungs was evaluated by using a seven-category scale (0–6 points), as previously described by Metz and Whishaw^86^ from all three videos. Metz and Whishaw´s scoring scale was divided into 3 categories: 0–2; 3–4; 5–6. Videos were then evaluated and the percentage of steps in each of the 3 categories was calculated. All the animals were pretrained before lesioning. The test was performed weekly for 16 weeks, starting 6 weeks after the SCI.

#### Mechanical pressure test (Von Frey test)

The Mechanical Pressure test was used to assess mechanical allodynia after the SCI and/or during treatment. The animals were placed in an enclosure with a metal mesh bottom and habituated there for 15 min. Von Frey rigid tip coupled with a force transducer (IITC Life Science, California, USA) was applied with a gradual increase of pressure to the footpad of the forepaw, until the animal withdrew its paw. The maximum pressure was recorded in grams. Five trials were performed on both hind paws. The trial was terminated if the animal failed to respond within 90 g. The average was set from three values after excluding the highest and lowest measurement. The test was performed 6 weeks after the SCI (before 4-MU treatment), after 8 weeks of rehabilitation (after 4-MU treatment), and at the end of the experiment.

#### Thermal test (plantar test)

The thermal test was used to assess a thermal hyperalgesia after the SCI and/or during treatment. The animals were placed in an acrylic box of the standard Ugo Basile test apparatus (Ugo Basile, Comerio, Italy) and habituated there for approximately 30 min. A mobile infrared-emitting lamp was then placed directly under the footpad of the hind paw, always in the same position. After placing the lamp, a thermal radiant stimulus was applied. The apparatus is connected to device what automatically recorded the time (in seconds) between the outset of the stimulus and the paw-withdrawal. Five trials were performed on both hind paws. The trial was terminated if the animal failed to respond within 30 s or wet oneself. The average was set from three values after excluding the highest and lowest measurement. The test was performed 6 weeks after the SCI (before feeding), after 8 weeks of rehabilitation (after feeding), and at the end of the experiment.

### Statistical analysis

Data processing and statistical analysis were performed using GraphPad Prism (GraphPad Software). To analyse the effect of 4-MU treatment on chronic SCI in rats, different statistical tests were used. The two-way *ANOVA* followed by Sidak’s multiple comparisons test was applied for astrogliosis, HABP, WFA, ACAN, 5-HT, synapsin intensity in SCI cohorts, BBB and Ladder Rung Walking test. The two-way *ANOVA* followed by Dunnett's test multiple comparisons test was applied intensity of HABP, number of cells enwrapped by PNNs within intact spinal cords, and for the gene expression of *has* genes. Different *post-hoc* tests for two-way ANOVA were used. Dunnett's test was used in order to test specifically the 4-MU effect and/or rehabilitation effect against a reference group (placebo without rehabilitation) in non-SCI cohorts results. Except for the two cases when the Tukey post hoc test was used for the PCR evaluation due to the reference of individual gene expression values to the untreated control without rehabilitation as well as for Maximum Speed test. Tukey test compares all possible pairs of means. For SCI cohort results, Sidak's multiple comparisons recommended for pairwise group comparisons were used. The total amount of GAGs was assessed by using the one-way *ANOVA* test followed by Dunnett's test multiple comparisons test. All the presented data in graphs were expressed as arithmetical means, with the standard error of the mean included. Significance as determined as followings: ns-not significant, *p < 0.05 **p < 0.01 ***p < 0.001 and ****p < 0.0001. Data were not assessed for normality. No test for outliers was conducted.

## Data Availability

The datasets used and/or analysed during the current study available from the corresponding author on reasonable request.

## Abbreviations

4-MU: 4-Methylumbelliferone
5-HT: 5-Hydroxytryptamine
ACAN: Aggrecan
ChAT: Choline acetyltransferase
ChABC: Chondroitinase ABC
CNS: Central nervous system
CS-GAGs: Chondroitin sulfate glycosaminoglycans
CSPGs: Chondroitin sulfate proteoglycans
HA: Hyaluronan
HABP: Hyaluronan binding protein
HASs: Hyaluronan synthases
UDP-GlcA: UDP-glucuronic acid
UGT: UDP-glucuronyl transferase
PNNs: Perineuronal nets
SCI: Spinal cord injury
WFA: *Wisteria floribunda* Agglutinin
