# Author Correction: Low oral dose of 4-methylumbelliferone reduces glial scar but is insufficient to induce functional recovery after spinal cord injury

**DOI:** 10.1038/s41598-023-50043-1

**Published:** 2024-01-08

**Authors:** Kateřina Štepánková, Milada Chudíčková, Zuzana Šimková, Noelia Martinez-Varea, Šárka Kubinová, Lucia Machová Urdzíková, Pavla Jendelová, Jessica C. F. Kwok

**Affiliations:** 1 Institute of Experimental Medicine, Czech Academy of Sciences, Vídeňská, 1083 Prague, Czech Republic; 2 Department of Neuroscience, Charles University, Second Faculty of Medicine, 15006 Prague, Czech Republic; 3 Institute of Physics, Czech Academy of Sciences, 182 21 Prague, Czech Republic; 4 Faculty of Biological Sciences, University of Leeds, Leeds, LS2 9JT UK

Correction to: *Scientific Reports* 10.1038/s41598-023-46539-5, published online 06 November 2023

The original version of this Article contained errors. As a result of incorrect figure assembly, in Figure 6D the image of Luxol Fast Blue staining for the +2 mm level was a duplication of the 0 mm level. Additionally, in Figure 7E the images were inadvertently switched for ‘Placebo’ and ‘4-MU’.

The original Figures 6 and 7 and their accompanying legends appear below.

**Figure 6:**
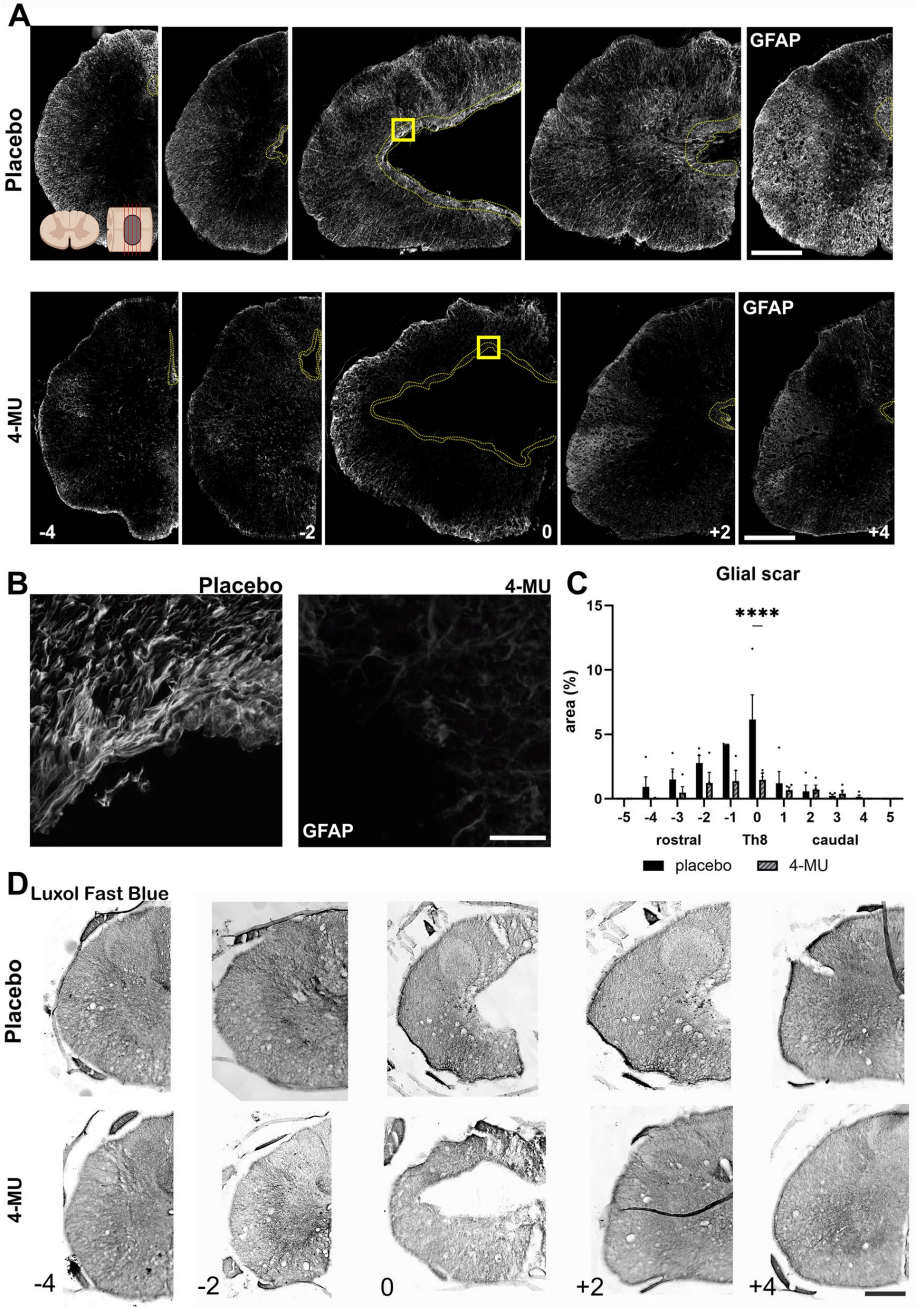
4-MU treatment reduced glial scar area surrounding the lesion site. (**A**) Representative fluorescent images showing lesion epicentre (0 mm), above (− 4, − 2 mm) and below (+ 2, + 4 mm) the lesion, stained for glial fibrillary acidic protein (GFAP) in placebo and 4-MU treated group with chronic spinal cord injury. Dotted lines show the border area of the lesion cavity in 4-MU treated group and GFAP positive area in placebo group. Scale bar: 200 µm. Diagram of uninjured spinal cord at top left showing the direction of the cross section in (**A**), created with BioRender.com; (**B**) magnified images (yellow square in **A**) showing structural change of the glial scar tissue after 4-MU treatment compared to placebo treated animals. Scale bar 30 µm; (**C**) bar graph showing area of the glial scar around the central cavity performed in the GFAP stained histochemical images using ImageJ software. Values are plotted as mean ± SEM; ****p < 0.0001 by two-way ANOVA, Sidak *post-hoc* test. (*n* = 4 animals per group). (**D**) Representative images of Luxol Fast Blue staining showing the lesion extension in a rostro-caudal direction. Scale bar 200 µm.

**Figure 7:**
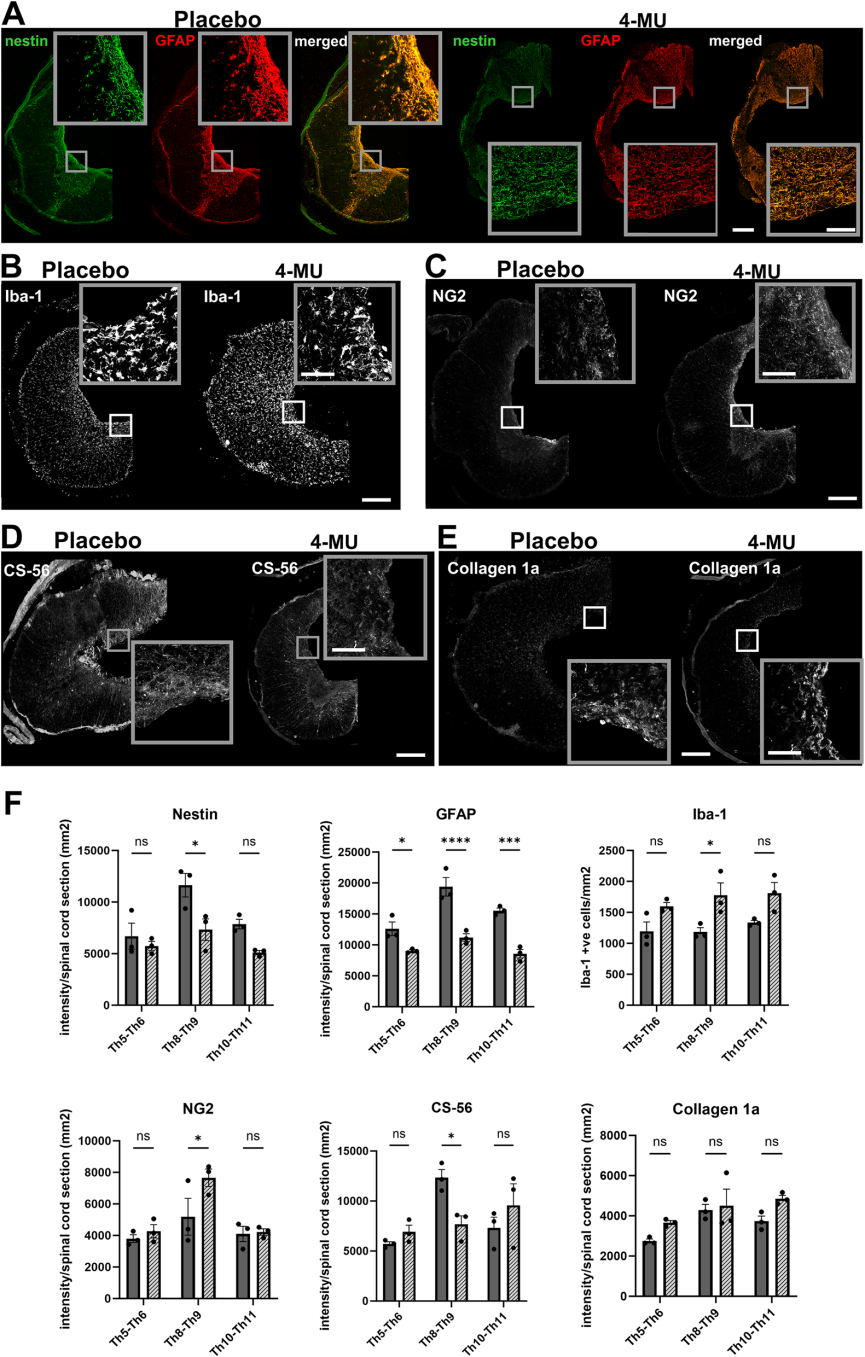
4-MU treatment leads to changes of cell and ECM composition around the lesion scar (Th8-9), above (Th5-6) and below (Th10-11) lesion. (**A**–**E**) Representative confocal images showing the 4-MU-mediated effect on scar-forming cells and components using different markers—(**A**) nestin and GFAP were used to visualise scar-forming astrocytes. (**B**) Iba-1 to visualise microglia/macrophages. (**C**) NG2 to visualise oligodendrocyte progenitor cells (OPCs). (**D**) CS-56 to examine the changes in CS sulfations. (**E**) Collagen 1a to visualise meninges and fibroblasts. All insets show magnified views of the staining. Scale bar 200 µm for the overview image and 50 µm for the insets. (**F**) Quantification of (**A**–**E**). Bar graphs show intensities per section throughout the spinal cord, except for Iba-1 staining where the number of Iba-1 positive cells per mm was counted. Individual data are shown with their mean ± SEM (n = 3 animals per group). p < 0.05, **p < 0.01, ***p < 0.001, ****p < 0.0001, by two-way ANOVA, Sidak's multiple comparison test.

The original Article has been corrected.

